# Low impact to fixed cell processing aiming transmission electron microscopy

**DOI:** 10.1590/0074-02760150433

**Published:** 2016-06

**Authors:** Ortrud Monika Barth, Marcos Alexandre Nunes da Silva, Debora Ferreira Barreto-Vieira

**Affiliations:** Fundação Oswaldo Cruz, Instituto Oswaldo Cruz, Laboratório de Morfologia e Morfogênese Viral, Rio de Janeiro, RJ, Brasil

**Keywords:** cell fixation, transmission electron microscopy, low impact, DENV

## Abstract

In cell culture, cell structures suffer strong impact due to centrifugation during processing for electron microscope observation. In order to minimise this effect, a new protocol was successfully developed. Using conventional reagents and equipments, it took over one week, but cell compression was reduced to none or the lowest deformation possible.

Cell and tissue are subjected to several impacts after fixation and when further processed for embedding in polymers, agar, gelatin and other media. Besides osmotic pressure and chemical attack, they may contract or swell according to the embedding medium applied (de Souza 2011).

Routinely (Barreto-Vieira et al. 2015),*Aedes albo-pictus* mosquito cell cultures (C6/36) and Vero cells infected with the four dengue virus (DENV) serotypes are fixed in buffered glutaraldehyde. The following steps require centrifugation, usually at least at 1,500 rpm. This attack results in morphological distortions and cell deformation (Barth 1992, 2000, 2010, Barth et al. 1996).

In order to minimise the impact caused to the cells by centrifugation, a new protocol was developed with success (Fig. 1).

**Fig. 1:**
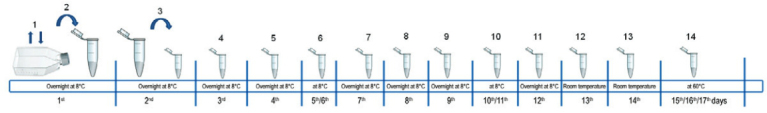
: protocol of low impact fixation of cells. (1-2) The culture medium was changed by cacodilate buffered 1% glutaraldehyde and aliquots of 1.5 mL of the cell suspension were kept overnight at 8ºC in 2 mL Eppendorf tubes; (3) a portion of the fixative was gently removed and the cell-fixative-suspension was transferred to smaller vials of 500 µL capacity; (4-5) the fixative was changed by 0.2 M cacodilate buffer added to 7% saccharose; (6) 0.2 M cacodilate buffer added to 7% saccharose was changed by 1% aqueous osmium tetroxide; (7) washing with distilled water and 0.2 M cacodilate buffer with 7% saccharose; (8) dehydration of the sample with 30% acetone; (9) dehydration with 50% acetone; (10) dehydration with 70% acetone; (11) dehydration with 90% acetone; (12) dehydration with 100% acetone and resin infiltration; (13) resin infiltration; (14) resin polymerization.

C6/36 cell monolayers were grown in 25 cm^2^ flaks for 24-48h at 28ºC using Leibovitz culture medium (L-15) supplemented with 1% non-essential aminoacids, 10% tryptose phosphate broth, 10% foetal bovine serum (Igarashi 1978). When the monolayer was established, cells were inoculated with 100 µL of DENV-4 suspension (title: 10^6.5^TCID_50_) and incubated during 1h at 28ºC for virus adsorption. Monolayers were subsequently grown in L-15 medium supplemented and kept at 28ºC. After incubation during six-12 days, cell monolayers presented around 30% of cytopathic effect.

The culture medium was changed to 5 mL of cacodilate (0.2 M pH 7.2) buffered 1% glutaraldehyde and after 5 min the flask was gently shaken in order to release the cells free. Aliquots of 1.5 mL of cell suspension were kept overnight at 8ºC in 2 mL Eppendorf tubes, so cells could precipitate. Centrifugation was never applied.

The following day, a portion of fixative was gently removed and the cell-fixative-suspension was transferred to 500 µL Eppendorf vials. The cells decanted onto the bottom of the vials again when left overnight in the refrigerator.

At the third day, the fixative was changed to 0.2 M cacodilate buffer added to 7% saccharose and the resuspended cells were left overnight in the refrigerator. This step was repeated the following day.

Post-fixation comprised 1% aqueous osmium tetroxide. The cell suspension was left for two days in the refrigerator. The cells changed to a darker colour and then utmost care had to be taken to remove the fixative. Cells were heavier at this time and precipitated more easily.

The next step consisted of washing sample with distilled water. After 1 h, the cells decanted onto the bottom. Then 0.2 M cacodilate buffer with 7% saccharose was added again and the sample was kept in the refrigerator overnight.

Dehydration of the sample intended inclusion in epoxy resin. It starts by replacing the buffer with 30% and 50% acetone, each one overnight, then with 70% acetone during two nights and 90% acetone in the refrigerator overnight.

Extending to room temperature, 100% acetone was replaced twice for 5 h.

Resin infiltration comprised three steps. Firstly a mixture of three parts of acetone and one part of resin was applied overnight, then one part of acetone and one part of resin, and at last one part of acetone and three parts of resin, both steps were also incubated overnight. Sample was kept in pure resin, which was changed the next day, for polymerization overnight.

Resin polymerization was executed during three days at 60ºC. After cooling down to room temperature, the resin blocks were mechanically released and no trimming was executed.

Although it took a longer time to conclude cell inclusion in resin when compared with traditional methodologies, preservation of cell structures showed none or very low cell compression, attributed to knife impact when cutting the resin blocks (Fig. 2A-B). Semithin sections of C6/36 cell pellets showed no cell compression (Fig. 2C-D). Ultrathin sections confirmed preserved cell structures (Fig. 2E) such as nucleus membranes, mitochondria, endoplasmic reticulum and ribosomes (Fig. 2F).

**Fig. 2:**
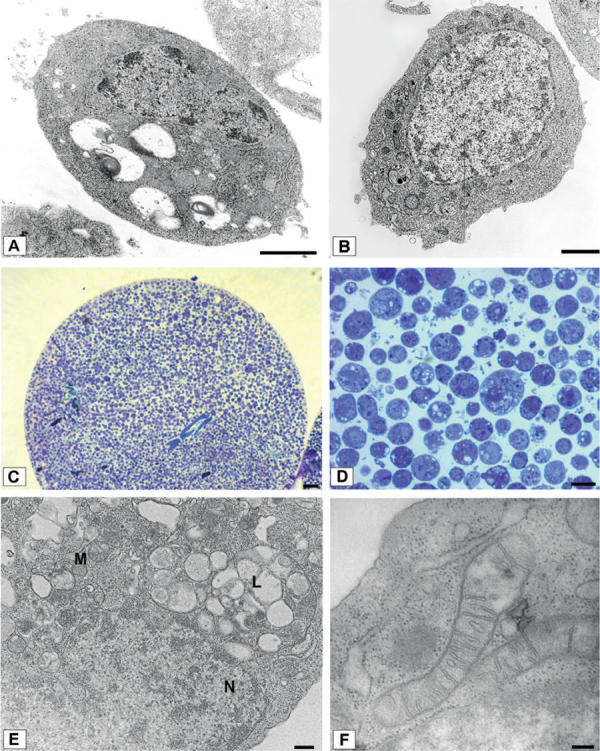
: (A) Conventional technique: ultrathin section of a compressed dengue virus-1 (DENV-1) infected C6/36 cell. It shows an ellyptical cell outline and laterally located nucleus (Bar = 1 μm); (B-F) the non-conventional technique; (B) ultrathin section of a non-compressed DENV-1 infected C6/36 cell. It shows a rounded cell and a centralised nucleus (Bar = 1 μm); (C) semithin section of a C6/36 cell pellet (Bar = 50 μm); (D) semithin section of C6/36 cells infected with DENV-4 (Bar = 10 μm); (E) ultrathin section of a C6/36 cell fragment showing nucleus (N), mitochondria (M), lysosome (L) (Bar = 200 nm); (F) ultrathin section of C6/36 cell mitochondria (Bar = 100 nm).

The technique presented above, aiming an extreme carefully processing of isolated cells grown in a liquid medium, may also be helpful in order to investigate cell compartment dimensions and its relation in spatial distribution. The proposed methodology minimises cell and organelle distortions when comparing with the conventional method that applies centrifugation.

## References

[B1] Barreto-Vieira DF, Takiya CM, Jácome FC, Rasinhas AC, Barth OM (2015). Secondary infection with dengue viruses in a murine model:
morphological analysis. Indian J Appl Res.

[B2] Barth OM, Cortes LMC, Farias J, Schatzmayr HG (1996). Ultrastructural aspects of the replication of dengue virus type 2
isolated in Brazil. Mem Inst Oswaldo Cruz.

[B3] Barth OM (2010). Atlas of dengue virus morphology and morphogenesis.

[B4] Barth OM (2000). Atlas of dengue viruses morphology and morphogenesis.

[B5] Barth OM (1992). Replication of dengue viruses in mosquito cell cultures - a model from
ultrastructural observations. Mem Inst Oswaldo Cruz.

[B6] Souza W (2011). Técnicas de microscopia eletrônica aplicadas às ciências biológicas.

[B7] Igarashi A (1978). Isolation of a singh’s Aedes albopictus cell clone sensitive to dengue
and Chikungunya viruses. J Gen Virol.

